# Improvement of Solder Joint Shear Strength under Formic Acid Atmosphere at A Low Temperature

**DOI:** 10.3390/ma17051055

**Published:** 2024-02-25

**Authors:** Siliang He, Jian Jiang, Yu-An Shen, Lanqing Mo, Yuhao Bi, Junke Wu, Chan Guo

**Affiliations:** 1 Key Laboratory of Microelectronic Packaging & Assembly Technology of Guangxi Education Department, School of Mechanical & Electrical Engineering, Guilin University of Electronic Technology, Guilin 541004, China; siliang_he@guet.edu.cn (S.H.); jian.jiang@cansemitech.com (J.J.); molanqing_guet@163.com (L.M.); biyh_guet@163.com (Y.B.); allenwjk@guet.edu.cn (J.W.); 2 Institute of Semiconductors, Guangdong Academy of Sciences, Guangzhou 510650, China; 3 Department of Materials Science and Engineering, Feng Chia University, Taichung 407, Taiwan; yashen@fcu.edu.tw

**Keywords:** solder joint, fluxless soldering, shear strength, formic acid, Sn-Bi solder

## Abstract

With the continuous reduction of chip size, fluxless soldering has brought attention to high-density, three-dimensional packaging. Although fluxless soldering technology with formic acid (FA) atmosphere has been presented, few studies have examined the effect of the Pt catalytic, preheating time, and soldering pad on FA soldering for the Sn-58Bi solder. The results have shown that the Pt catalytic can promote oxidation–reduction and the formation of a large pore in the Sn-58Bi/Cu solder joint, which causes a decrease in shear strength. ENIG (electroless nickel immersion gold) improves soldering strength. The shear strength of Sn-58Bi/ENIG increases under the Pt catalytic FA atmosphere process due to the isolation of the Au layer on ENIG. The Au layer protects metal from corrosion and provides a good contact surface for the Sn-58Bi solder. The shear strength of the Sn-58Bi/ENIG joints under a Pt catalytic atmosphere improved by 44.7% compared to using a Cu pad. These findings reveal the improvement of the shear strength of solder joints bonded at low temperatures under the FA atmosphere.

## 1. Introduction

Soldering is a commonly used technique in microelectronics for connecting chips and substrates [1,2,3]. The solder joints provide the necessary electrical, thermal, and mechanical properties for a reliable connection [4,5,6]. During the traditional soldering process, organic flux is applied to metal surfaces to remove dirt and oxide, enhancing the soldering reliability. However, if the flux residues existed in the solder joint, it can cause severe corrosion and the weakening of the soldering strength [7,8,9,10]. With the miniaturization of solder joints in three-dimensional integrated circuits, it has become more challenging to eliminate the flux, and its impact on the interconnection is becoming increasingly significant [11,12]. Therefore, considerable attention has been given to the development of fluxless soldering processes. One promising method involves replacing traditional flux with a reduction atmosphere.

A formic acid (FA) atmosphere is a promising reduction agent due to its ability to reduce at a temperature range of 150–250 °C, which is suitable within the melting temperature of most solders. IBM Canada Laboratory used an FA atmosphere to achieve fluxless chip-level packaging [13]. The interconnection with a 10 μm fine pitch achieved within an FA atmosphere has been revealed [14]. Furthermore, the realization of Cu/SiO_2_ hybrid bonding at a low temperature is accomplished through the optimization of the process of Ar/O_2_ plasma activation and an FA solution immersion [15]. These studies have shown that using FA can effectively reduce microelectronic metal oxide materials, which are suitable for high-density, small-size packaging structures. Typically, the effectiveness of the FA atmosphere in reducing materials can be improved with an increase in the soldering temperature. However, in previous studies, Sn steaming occurred under an FA atmosphere of 210 °C and induced the dispersion of the Sn, which may cause the short circuit problems of electronic packaging [16]. Hence, FA soldering is more suitable at low-temperature conditions. 

The Sn-58Bi eutectic solder with a melting temperature of 138 °C possesses great mechanical strength and suitable ductility [17]. Thus, it is normally used for soldering below 200 °C to resist the destruction of thermal warpages [18]. Also, the Sn-58Bi can be a favorable candidate for low-temperature fluxless soldering, especially for FA soldering [19].

However, because few studies reveal the soldering process within an FA atmosphere at temperatures below 200 °C, the FA reaction with Sn-58Bi has not been understood well, especially since the activity of the FA gas phase molecules decreases as the temperature decreases. On the other hand, although Pt is a good catalytic to enhance FA soldering [20,21], studies to investigate the FA soldering of Sn-58Bi with the Pt catalytic are few.

This study aims to investigate the effect of an FA atmosphere on the soldering reaction of an Sn-58Bi with copper and ENIG substrates. In addition, the study of Pt as a catalytic to accelerate FA in low-temperature soldering is revealed.

## 2. Materials and Methods

### 2.1. Materials

For this study, a Cu-OSP PCB with a copper pad 480 μm in diameter was used. The solder used was an Sn-58Bi with a diameter of 760 μm. A PCB conducted another set of experiments with an ENIG surface finish. Before FA soldering, hydrochloric acid and anhydrous ethanol were used to clean the surface dirt from the soldering pad.

### 2.2. Experiment

**Figure 1:**
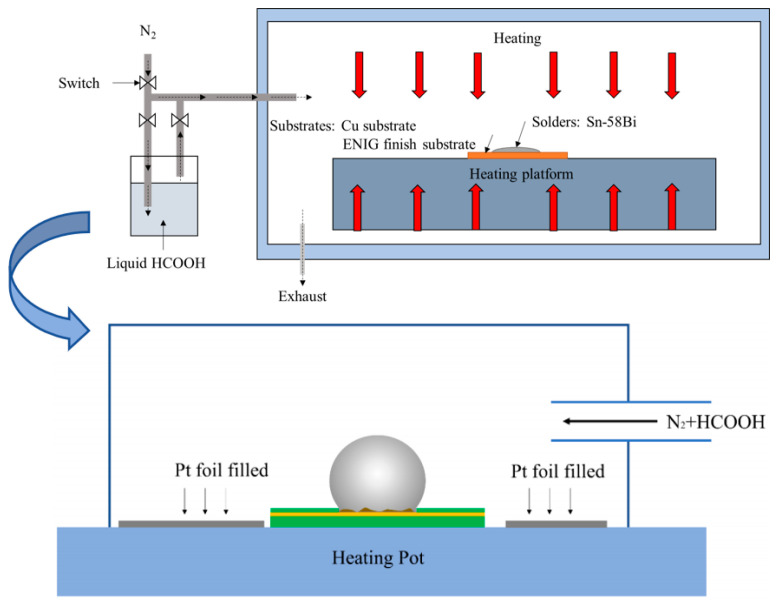
Schematic diagram of the FA atmosphere passing into the reflow equipment (Beijing, China) and the Pt catalytic FA atmosphere process.

The preheating temperature of the Sn-58Bi solder joint was set to 120 °C for 1 min, and the reflow peak temperature was 200 °C for 2 min. The heating rate was 2.5 °C/s. To examine the effect of the Pt catalytic on the shear strength of the solder joints by FA soldering, a Pt strip was attached during the heating stage (Figure 1).

To investigate the effect of preheating times on the Sn-58Bi/Cu joint shear strength in an FA atmosphere, the samples were preheated at 120 °C for 2, 5, and 10 min, followed by a peak temperature of 200 °C for 2 min.

### 2.3. Shear Test

**Figure 2:**
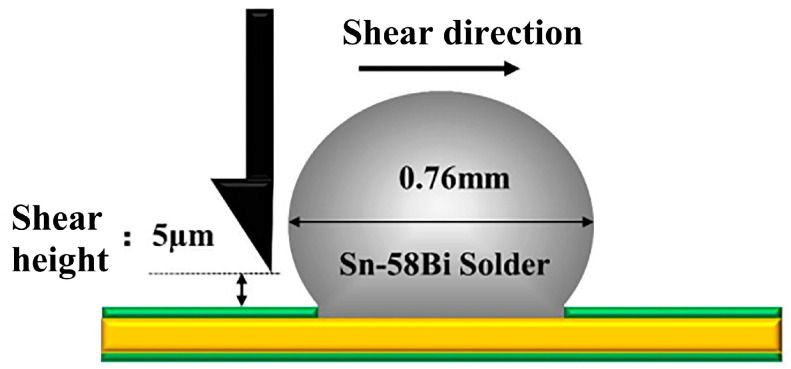
Schematic diagram of the low-speed shear test.

To determine the soldering strength of the solder joints after reflow, a low-speed shear test was performed. The MFM1200 push-tension tester (Hongkong, China) was used for the test, and the testing schematic is shown in Figure 2. The shear rate was 600 µm/s, and the height was maintained at 5 µm from the pad. Once the test was complete, the computer generated a shear curve, which was then used to calculate the result through the shear strength formula. A solder pad measuring 480 μm was used as the soldering area to calculate the shear strength.

## 3. Results and Discussion

### 3.1. Microstructure and Shear Strength of Sn-58Bi/Cu Solder Joints under FA Atmosphere at 200 °C

**Figure 3:**
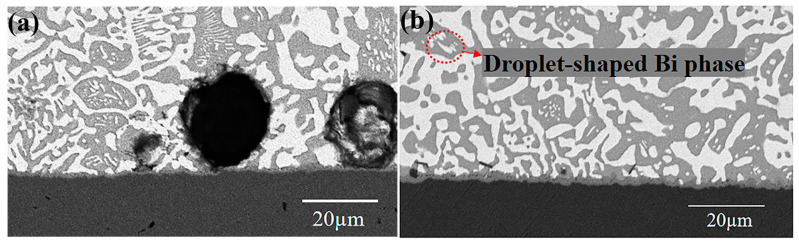
BSE image of Sn-58Bi/Cu solder joint under FA atmosphere: (**a**) Sn-58Bi/Cu solder joint after reflow through FA atmosphere, (**b**) the scattered droplet-shaped Bi phase.

From Figure 3a, a thin IMC layer has formed at the interface between the Sn-58Bi and the Cu pad after the FA reflow. The IMC appears scalloped, continuous, and uniform, indicating a stronger metallurgical bonding of the joint. However, there are visible pores above the IMC layer. It is suspected that the interfacial reaction with the FA atmosphere generated some metal formate and some gas to form the pores during reflow. When the temperature rises above 150 °C, Sn and Cu formates decompose into Sn, Cu, CO_2_, and H_2_ [22]. The grey area above the interface is the Sn-rich phase, and the white one is the Bi-rich phase. A less regular lamellar structure has formed between the Sn and Bi-rich phases. The droplet-like structures of the Bi-rich phase are inconsistent with the lamellar structure, most of which exist in the Sn-rich phase. When the Sn–Bi alloy solidifies, the Sn—Bi solder experiences a certain degree of supercooling below the eutectic point. In a short time, the Sn-rich and Bi-rich phases create and form an irregular lamellar eutectic structure. However, in the subsequent cooling process, those small Bi grains in the Sn-rich phase precipitate out due to the low solubility at low temperatures, thus forming the scattered droplet-shaped Bi phase in the Sn phase in Figure 3b [23].

**Figure 4:**
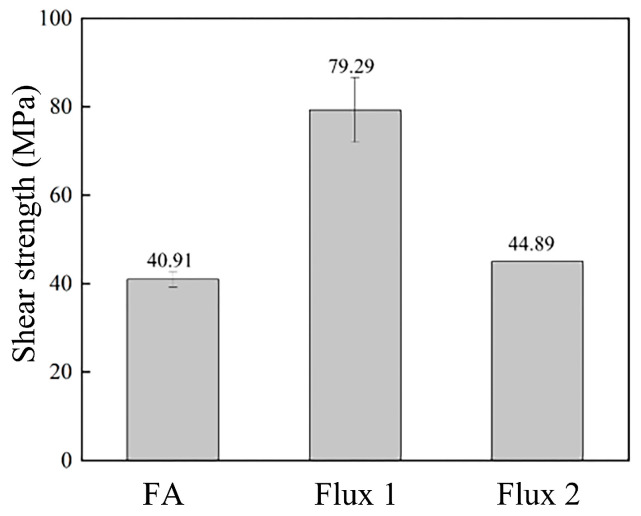
Shear strength of Sn-58Bi/Cu solder joints after reflow of FA atmosphere process and Flux process.

In order to confirm the reliability of the solder joint in the FA atmosphere, the strength of the other two processes was compared. In Figure 4, the shear strength of the FA soldering joint is 40.91 MPa. In comparison, the shear strength of a solder joint using soldering Flux 1 is 79.29 MPa, and one using soldering Flux 2 [24] is 44.89 MPa. The results show that the shear strength of FA soldering in this study is lower than that of Flux 1 but very similar to that of Flux 2, which is used in the literature. Therefore, it can be concluded that the FA soldering of this study performs with a reliable soldering strength.

**Figure 5:**
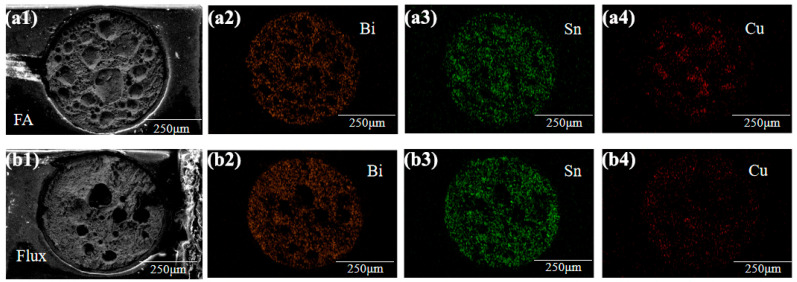
EDS surface scanning energy spectrum of Sn-58Bi after reflow in FA atmosphere process and Flux process: (**a1**) Fracture SEM of the FA atmosphere, (**a2**) the EDS mapping of Bi element in (**a1**), (**a3**) the EDS mapping of Sn element in (**a1**), (**a4**) the EDS mapping of Cu element in (**a1**); (**b1**) Fracture SEM of the Flux process, (**b2**) the EDS mapping of Bi element in (**b1**), (**b3**) the EDS mapping of Sn element in (**b1**), (**b4**) the EDS mapping of Cu element in (**b1**).

**Figure 6:**
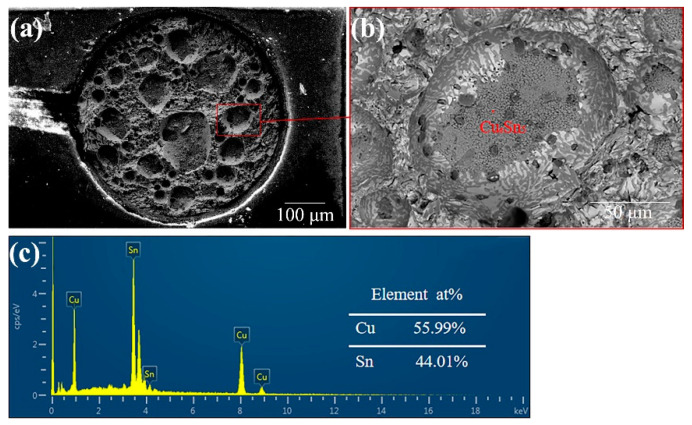
Sn-58Bi solder joint fracture surface and hole enlargement are formed by FA atmosphere process, (**a**) section SEM map, (**b**) section enlarged BSE image, (**c**) the EDS point energy spectrum in (**b**).

The morphology and elemental distribution on the fracture surface of the joints reflowed under the FA atmosphere are shown in Figure 5. Different colored elements appear in the diagram: orange Bi, green Sn, and red Cu. The main elements are Sn and Bi, indicating that fracture occurs in the Sn-58Bi solder either in an FA atmosphere or with flux. In Figure 6a, the red dotted line indicates the areas of the pores that are larger than 50 µm. Table 1 shows the percentage porosity on the fracture surfaces of the solder joints. The percentage porosity of FA soldering is 32.3%, while that using soldering flux is 5.4%. This means that FA soldering forms large pores which lead to a lower shear strength than flux soldering. In Figure 6b,c, an EDS elemental analysis confirms that the holes are formed above the IMC of Cu_6_Sn_5_, which also weakens the FA soldering interface. These results may be because Flux 1 is designed for low-temperature soldering and to remove oxides at a temperature below 200 °C. FA soldering at 200 °C cannot reduce oxides completely, causing large pores to form during reflow. Although the performance of low-temperature FA soldering is worse than that of flux soldering, the former still shows good soldering strength. Improving the process conditions of FA soldering can promote the development of fluxless soldering.

**Figure 7:**
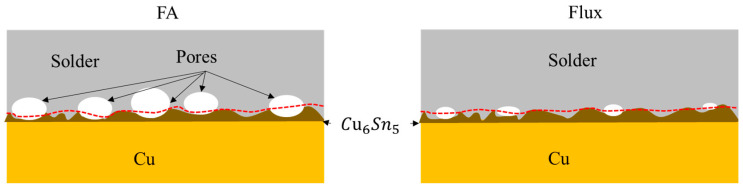
Schematic diagram of fracture path after Sn-58Bi/Cu under the FA soldering (**left**) and the flux soldering (**right**). The red dashed line is the schematical line of fracture area.

Combined with Figure 6 and Table 1, it is speculated that the fracture of Sn-58Bi/Cu in the FA atmosphere is mainly due to the pores generated during soldering, which extend along the whole side wall, and the fracture is located above the IMC. The fracture of Sn-58Bi/Cu in the Flux process at the junction of the solder and the IMC is a brittle fracture. The fracture path of the solder under different processes is shown in Figure 7.

### 3.2. Shear Strength of Sn-58Bi/Cu Solder Joint Formed under Pt Catalytic FA Atmosphere

**Figure 8:**
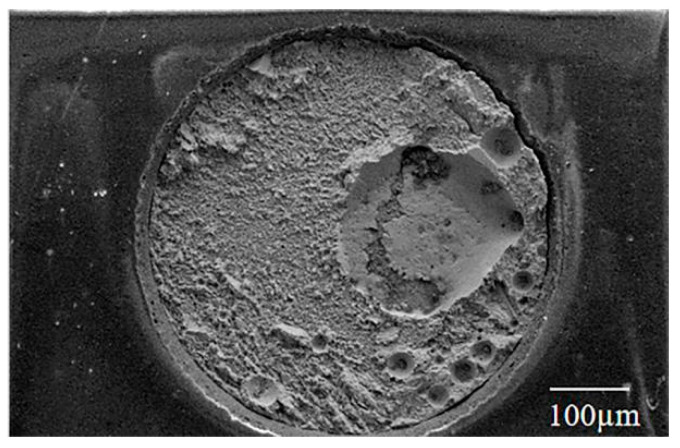
SEM fracture image of Sn-58Bi/Cu solder joint after reflow of Pt catalytic FA atmosphere process.

The shear strength of the FA soldering joint with the Pt catalytic as tested by the low-speed shear test, is shown in Table 2. Figure 8 shows the fracture morphology of the FA soldering joint with the Pt catalytic as tested by the low-speed shear test. The joint with the Pt catalytic has a lower porosity ratio of 23.5% compared to the joint without it, as summarized in Table 3. This improvement can be attributed to the promotion of the Pt catalytic with oxidation–reduction by the FA atmosphere, and the schematic of the catalytic reaction is shown in Figure S1 in Supplementary Materials. 

However, the shear strength of the Sn-58Bi/Cu solder joint is 39.19 MPa, lower than that of the solder joint without the Pt catalytic. According to Figure 8, the fracture mode of the FA soldering with the Pt catalytic is not significantly changed, but a large pore that formed in the solder of the joint reduced the soldering strength. The Pt catalytic can promote the decomposition of the oxidation–reduction by FA, and the H_2_ and CO_2_ produced by the reduction process cannot be discharged, causing the formation of the large pore in the solder. Therefore, eliminating gases produced by oxidation–reduction during FA soldering with the Pt catalytic is crucial.

### 3.3. Shear Strength of Sn-58Bi/Cu Solder Joint Formed Using Different Preheating Times under FA Atmosphere

Reflow time and temperature are important factors for soldering. Still, previous studies have often focused on the influence of peak time and peak temperature on the wettability of the solder on the pad. The latest study found that increasing the peak temperature in FA soldering produces the Sn steam before the solder melts at a low temperature [16]. The interfacial reaction and wettability can be improved by the reaction of the Sn steam with Cu because of a primary IMC formation before the melting of the solder. The longer preheating time can improve the interfacial reaction, but other reported studies are seldom about this issue for Sn-58Bi/Cu. Table 4 summarizes the different preheating times (2, 5, and 10 min) for the FA soldering of Sn-58Bi/Cu joints at a temperature of 120 °C. Processes 1, 2, and 3 represent the set process parameters with preheating times of 2, 5, and 10 min, respectively.

**Figure 9:**
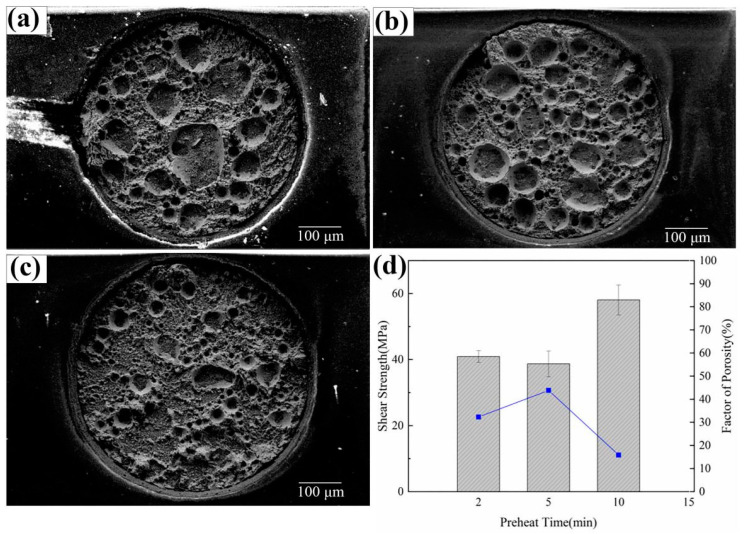
SEM and corresponding shear strength and porosity changes of Sn-58Bi/Cu solder joints formed at different preheating times under FA atmosphere process. Preheating time: (**a**) 2 min, (**b**) 5 min, (**c**) 10 min; (**d**) the shear strength at different preheating times (column chart) and its corresponding porosity (line chart).

Figure 9 shows the fracture morphology and shear strength of the solder joint with each preheating time. Figure 9a shows the fracture surface of a sheared joint with a preheating time of 2 min and the pores formed in this joint. The porosity cannot be effectively improved by preheating for 5 min (Figure 9b). The porosity ratios of the latter and the former are approximately 42% and 32% respectively. However, when the preheating time is extended to 10 min, the porosity ratio on the fracture surface of the joint decreases significantly (Figure 9c). The fraction of porosity of the 10 min preheat time is 15.8%. Such porosity changes also result in changes in soldering strength. The soldering strength of Sn-58Bi/Cu joints with preheating times of 2 min and 5 min is 42 MPa and 38 MPa, respectively, while the joints with a preheating time of 10 min present the highest soldering strength of 58 MPa. This not only proves that porosity has a great effect on the reliability of FA soldering, but it also shows that increasing preheating time can significantly reduce the formation of pores in the Sn-58Bi/Cu joint. FA can remove the oxide on the metallic surfaces to improve soldering, and appropriately extending the preheating time can provide sufficient time for oxide–reduction by the FA atmosphere. The reduction of soldering interfacial pores enhances the soldering strength. Moreover, the extension of the preheating time can reduce the thermal stresses generated during the soldering process.

Although increasing the preheating time is effective for improving FA soldering, the time required for the process is often a key factor in practical applications. It is possible to significantly reduce the amount of oxide present in soldering materials with effective measures. Consequently, the next section will exhibit the results of Sn-58Bi/ENIG assembled by FA soldering, Flux soldering, and FA soldering with Pt catalytic.

### 3.4. Shear Strength of Sn-58Bi/EING Solder Joint Formed under Pt Catalytic FA Atmosphere

**Figure 10:**
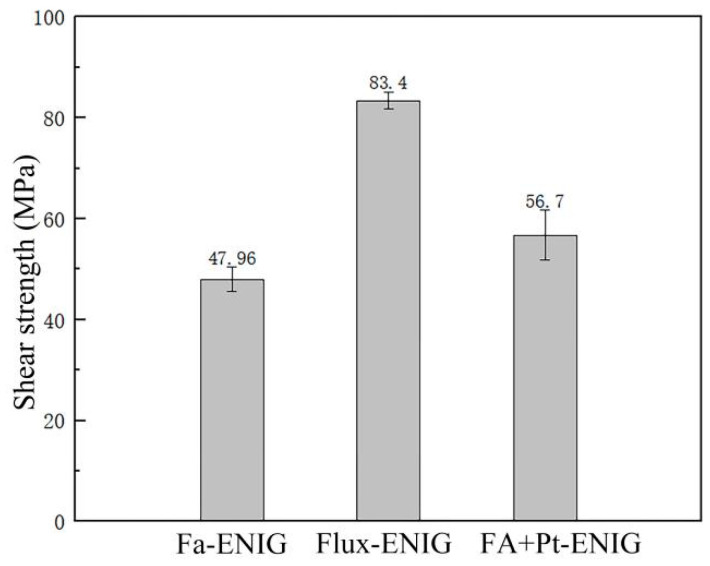
Sn-58Bi/ENIG solder joint shear strength formed by FA atmosphere process, flux process, and Pt catalytic FA atmosphere process.

To minimize the formation of pores, it is crucial to address the issue of copper pad oxidation. One effective way to prevent oxidation of the substrate or solder joint is by using ENIG instead of copper pads. Previous studies reported ENIG has great wettability with SAC solder, and the Ni layer can resist the rapid reaction of Cu with Sn to form thick interfacial IMC [25,26]. As shown in Figure 10, the enhancement in the soldering strength of Sn-58Bi by ENIG is significant. The shear strength of Sn-58Bi/ENIG by FA + Pt is 56.7 MPa, which is 44.7% higher than that of Sn-58Bi/Cu (39.19 MPa). Additionally, the shear strength of the Sn-58Bi/ENIG by FA soldering without the Pt catalytic is 47.96 MPa, which is increased by 17.2%. The flux soldering by ENIG enhancement is approximately 4%. Therefore, the ENIG enhancement to FA soldering is effective.

**Figure 11:**
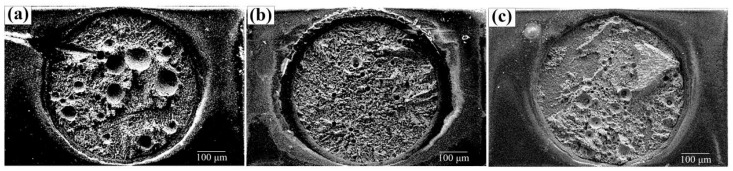
(**a**) FA atmosphere process, (**b**) flux process, and (**c**) Pt catalytic FA atmosphere process.

The results as shown in Figure 11, indicate that using the ENIG pad instead of the Cu pad causes a reduction in the porosity of the solder joint by the three processes. Additionally, it reduces the size of the pores formed at the interface. This can be attributed to the isolation of the Au layer on ENIG. This layer protects the underlying metal from oxidation and corrosion and provides an excellent contact surface for the Sn-58Bi solder. However, FA soldering alone cannot effectively reduce the oxide in a short period of time, so the performance difference between ENIG and Cu is only 17%. The Pt catalytic FA atmosphere process increases the strength more than the FA atmosphere process by using ENIG pads because the Pt catalysis makes FA reduce the surface oxide of the Sn-58Bi solder joint more fully.

**Figure 12:**
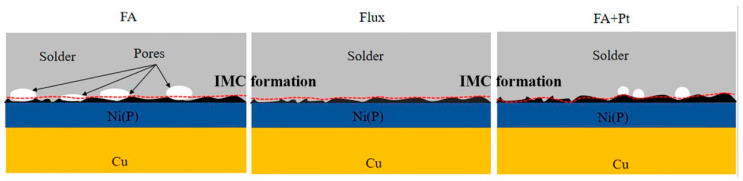
Schematic diagram of fracture path after Sn-58Bi/ENIG under the FA atmosphere process (**left**), the flux process (**middle**), and the Pt catalytic FA atmosphere process (**right**). The red dashed line is the schematical line of fracture area.

Figure 12 shows the fracture morphologies of the joints by the three different processes. The locations of fracture occurrence in the joints are similar: near the interfacial IMC. However, the percentage porosity in the joints of flux soldering and FA soldering with Pt is much lower than that of FA soldering. The percentage porosity of the solder joint determined the soldering strength. Therefore, this study suggests that the coupling of FA and a Pt catalyst can perform a fluxless joint with great soldering strength.

## 4. Conclusions

In this study, the shear strength of Sn-58Bi/Cu solder joints was studied under the FA atmosphere process, the flux process, and the Pt catalytic FA atmosphere process at low temperatures. The results are as follows:

(a) Pores appear in Sn-58Bi/Cu solder joints, and the microstructure of the flux process interface is relatively complete. The shear strength of Sn-58Bi/Cu solder joints is relatively poor by FA soldering.
(b) The shear strength of Sn-58Bi/Cu under the Pt catalytic FA atmosphere is lower than that of Sn-58Bi/Cu in the FA atmosphere. Although the Pt catalytic FA atmosphere process is more efficient in reducing metal oxides, the formation of large pores above the bonding interface is greater, leading to a further decline in mechanical properties.
(c) Under the FA atmosphere, by keeping other reflow process parameters unchanged and appropriately extending the preheating time (10 min), the porosity of Sn-58Bi/Cu (15.8%) can be significantly reduced and the shear strength increased to 58 MPa because the Cu pad is fully restored.
(d) The shear strength of Sn-58Bi/ENIG joints under the FA atmosphere process and the Pt catalytic FA atmosphere process was 47.96 MPa and 56.71 MPa, respectively, which improved the mechanical properties by 17.2% and 44.7%, respectively, compared with the Cu pad. The Sn-58Bi/ENIG fracture interface showed a great improvement in porosity compared to the Sn-58Bi/Cu fracture interface.

## Figures and Tables

**Table 1:** Percentage porosity of the Sn-58Bi/Cu fracture interface formed by the FA process and Flux process.

Process	Percentage Porosity (%)
FA	32.3
Flux	5.4

**Table 2:** Shear strength of the Sn-58Bi/Cu under FA atmosphere and Pt catalytic FA atmosphere.

Process	Shear Strength (MPa)
FA	40.91
FA + Pt	39.19

**Table 3:** Percentage porosity of the Sn-58Bi/Cu fracture interface between the FA atmosphere and Pt catalytic FA atmosphere.

Process	Percentage Porosity (%)
FA	32.3
Pt catalytic	23.5

**Table 4:** Different preheating time processes of Sn-58Bi Cu under FA atmosphere.

Process	1	2	3
Preheat temperature (°C)	120	120	120
Preheat time (minute)	2	5	10
Peak temperature (°C)	200	200	200
Peak time (minute)	2	2	2
Ramp rate (°C/s)	1	1	1

## Data Availability

The data are not publicly available due to privacy.

## Supplementary Materials

The following supporting information can be downloaded at https://www.mdpi.com/article/10.3390/ma17051055/s1, Figure S1: Schematic diagram of Pt catalytic principle.
