# Supplementary figures and images for: Improvement of Solder Joint Shear Strength under Formic Acid Atmosphere at A Low Temperature
Source: Materials (Basel). 2024 Feb 25;17(5):1055. doi: 10.3390/ma17051055 (PMC10934809; doi:10.3390/ma17051055)

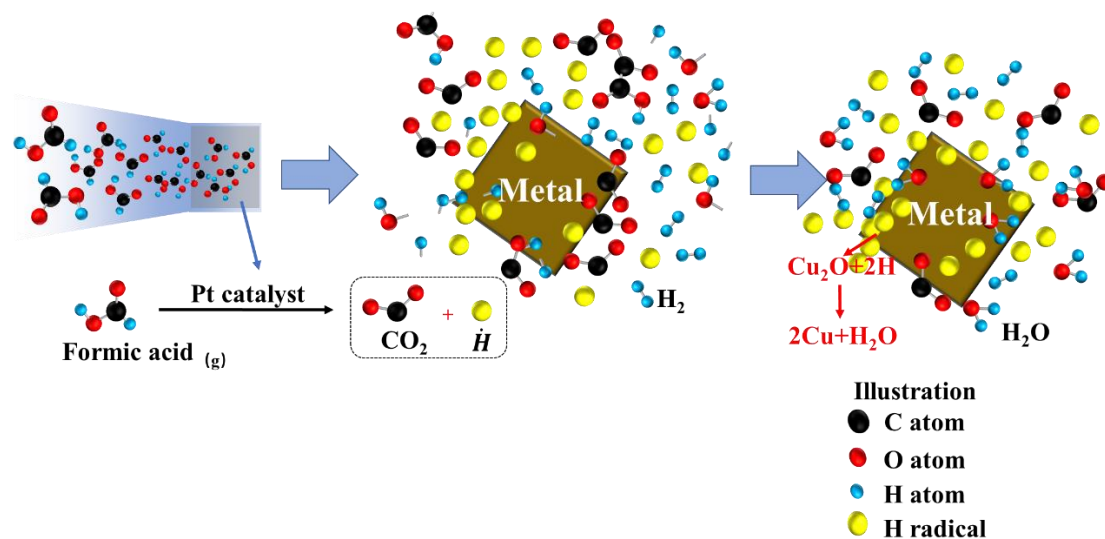

Fig. S1. Schematic diagram of Pt catalytic principle.

Supplement: Supplementary file 1 [file materials-17-01055-s001.zip › materials-2828482-supplementary.pdf]
